# An epidemiological study of avian influenza A (H5) virus in nomadic ducks and their raising practices in northeastern Bangladesh, 2011‐2012

**DOI:** 10.1111/irv.12438

**Published:** 2017-01-02

**Authors:** Shamim Sarkar, Salah Uddin Khan, Andrea Mikolon, Mohammad Ziaur Rahman, Jaynal Abedin, Nord Zeidner, Katherine Sturm‐Ramirez, Stephen P. Luby

**Affiliations:** ^1^ Programme on Emerging Infections (PEI)Infectious Diseases Division (IDD)icddr,b, DhakaBangladesh; ^2^ College of Public Health and Health Professionals and Emerging Pathogen InstituteUniversity of FloridaGainesvilleFLUSA; ^3^ California Department of Food &AgricultureOntarioCAUSA; ^4^ Centers for Disease Control and Prevention (CDC)AtlantaGAUSA; ^5^ Center for Innovation in Global HealthStanford UniversityStanfordCAUSA

**Keywords:** avian influenza A (H5) virus, egg yolk, H5‐antibodies, nomadic duck, wild waterfowl and Bangladesh

## Abstract

**Background:**

In Bangladesh, nomadic duck flocks are groups of domestic ducks reared for egg production that are moved to access feeding sites beyond their owners’ village boundaries and are housed overnight in portable enclosures in scavenging areas. The objectives of this study were to measure the prevalence of influenza A virus RNA and H5‐specific antibodies in nomadic ducks and to characterize nomadic duck raising practices in northeastern Bangladesh.

**Methods:**

We tested duck egg yolk specimens by competitive ELISA to detect antibodies against avian influenza A (H5) and environmental fecal samples by real‐time reverse‐transcription polymerase chain reaction (rRT‐PCR) to detect influenza A virus RNA and H5 subtype.

**Results:**

The median age of the ducks was 24 months (range: 8‐36 months) and the median flock size was 300 ducks (range: 105‐1100). Of 1860 egg yolk samples, 556 (30%, 95% confidence interval (CI): 28‐32) were positive for antibodies against H5 and 58 flocks (94%) had at least one egg with H5‐specific antibodies. Of 496 fecal samples, 121 (24%, 95% CI: 22‐29) had detectable influenza A RNA. Thirty‐three flocks (53%) had at least one fecal sample positive for influenza A RNA.

**Conclusions:**

Nomadic ducks in Bangladesh are commonly infected with avian influenza A (H5) virus and may serve as a bridging host for transmission of avian influenza A (H5) virus or other avian influenza A viruses subtypes between wild waterfowl, backyard poultry, and humans in Bangladesh.

## 1 Introduction

Waterfowl are a natural reservoir for all subtypes of influenza A viruses.^1^, ^2^ Highly pathogenic avian influenza A (H5) viruses in domestic ducks may result in asymptomatic, subclinical, or clinical infections, and asymptomatic ducks often shed the viruses through feces and respiratory droplets.^3^, ^4^ In many Asian countries, farmers herd scavenging ducks from one feeding ground to another through the year and these practices can contribute to the spread of influenza A (H5) viruses.^5^, ^6^, ^7^ Allowing contact between domestic ducks, wild waterfowl 5, 6, 8 and other poultry and animal species, poses risks for spreading of influenza A (H5) viruses.^5^, ^6^, ^9^

Northeastern Bangladesh, with its high intensity domestic duck raising and an agroecological landscape with extensive interface between large water bodies and rice fields, acts as an important site for interaction between wild waterfowl and domestic ducks^10^ especially during the winter. Highly pathogenic avian influenza (HPAI) A(H5N1) virus clade 2.3.2.1 has been circulating among poultry in Bangladesh since 2011.^11^ However, HPAI A (H5N1) virus clade 2.3.2.1 was previously isolated from wild waterfowl in Bangladesh in 2010.^12^

Bangladesh had an estimated 41 million ducks in 2009.^13^ Ducks are mainly raised for egg production in Bangladesh^14^ where they provide an important source of protein, self‐employment, and livelihood for rural people.^15^, ^16^ Nomadic duck raising in Bangladesh occurs mainly in low‐lying areas around large water bodies^17^ and adjacent to harvested rice fields which provide feed and serve as sites of interaction between domestic ducks and wild waterfowl. Netrokona, Sunamganj, Noakhali, Habiganj, and Moulvibazar districts^18^ are the main nomadic duck raising areas in the country.^19^, ^20^ In these districts, nomadic ducks have opportunities for contact with wild waterfowl since they both scavenge in the same water bodies, whereas backyard ducks remain near their owners’ household premises.

Since March 2007, over 500 outbreaks of HPAI A(H5N1) virus have been reported in chickens in Bangladesh.^18^ Live bird market surveillance has identified HPAI A(H5N1) virus in apparently healthy ducks in Bangladesh since 2007,^21^ and in 2011, there was an outbreak with unusual duck mortality due to HPAI A(H5N1) 2.3.2.1a virus in northeastern Bangladesh.^22^

We conducted a study to measure the prevalence of H5‐specific antibodies and influenza A virus RNA in nomadic ducks and to characterize nomadic duck raising practices in northeastern Bangladesh. This study will help determine whether nomadic ducks are a substantial reservoir of avian influenza A (H5) viruses and describe the nomadic duck raising practices associated with AIV carriage that could be amenable to culturally appropriate, effective, and affordable intervention.

## 2 Methods

### 2.1 Study site and population

We selected Mohanganj subdistrict of Netrokona District in the northeastern part of Bangladesh because it has large bodies of water in low‐lying areas and domestic ducks raised in the nomadic system^18^ that interact with wild waterfowl during winter (November‐February).^23^ There were an estimated 2.5 million domestic ducks in the Mohanganj subdistrict in 2006.^18^ A large number are reared nomadically for egg production and the rest are backyard ducks (Department of Livestock Services). Ongoing live bird market surveillance frequently identifies H5N1 among domestic ducks in Mohanganj.^21^ Five months before our study began, a reported outbreak of HPAI A (H5N1) with high mortality occurred among poultry (ducks, geese, and chickens) in this study area.^22^

### 2.2 Study design

From December 2011 through February 2012, we conducted a cross‐sectional study of 62 nomadic duck flocks within Mohanganj to collect duck eggs and swab samples from fresh fecal droppings and interview flock owners. We chose egg yolk samples to detect antibodies against avian influenza A (H5) instead of serum samples because blood collection in egg‐laying ducks has practical difficulties: catching and collecting blood samples from laying ducks is stressful to the ducks which causes financial losses through reduced egg production.^24^ Others studies demonstrated that egg yolk is a good alternative source for the detection of antibodies of avian influenza viruses in laying hens and ducks.^24^, ^25^, ^26^ Another study found a high correlation between H5 antibodies in egg yolk and serum samples.^27^

**Figure 1:**
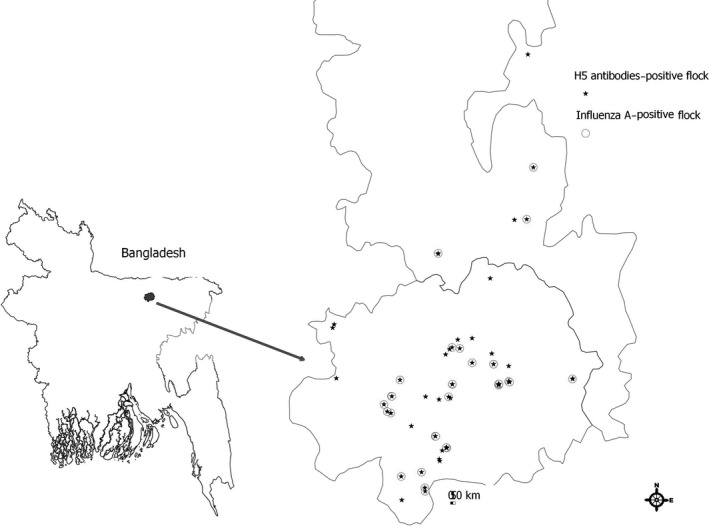
Location to detect avian influenza A RNA and with H5 antibody positive among nomadic duck flocks within northeastern Bangladesh, December 2011 to March 2012

We defined nomadic duck flocks as groups of domestic ducks reared for egg production that are moved to access feeding sites beyond their owners’ village boundaries and are housed overnight in portable enclosures in scavenging areas (Figure 1).

### 2.3 Sample size

The (HPAI) virus (H5N1) is endemic in poultry in five countries: Bangladesh, China, Egypt, Indonesia, and Vietnam.^28^ Our sample size calculation was based on the prevalence of antibodies to H5 in duck flocks in Indonesia in 2007‐2008. The flock‐level prevalence of antibodies to H5 was 19.5%.^6^ To determine the number of nomadic duck flock, we assumed flock‐level prevalence of antibodies to H5 of 20%, a precision of 10%, and a 95% confidence interval (CI). Our estimated number of flocks needed for this study was 62.

To determine the number of egg samples necessary to detect an assumed H5 antibody prevalence of 2.6%^6^ with 1% precision and a 95% CI, we multiplied the calculated sample size by the assumed design effect of 2^29^ to account for the cluster sampling strategy. Our calculated egg sample size was 1860. To determine the number of eggs sampled within a flock to detect H5 antibodies, we assumed 10% expected prevalence with 95% CI. Our estimated egg sample was 30 within a flock.

### 2.4 Sampling

We collected available information about nomadic duck flocks from the Mohanganj Upazila Government Veterinary Hospital and from the flock owners using the chain referral technique.^30^ We prepared a list of 90 nomadic duck flocks with at least 100 egg‐laying ducks in each flock and assigned a unique number to each flock owner. We generated a random number using Microsoft Excel to select 62 nomadic duck flocks from this list. Among them, 56 flock owners agreed to participate; the remaining six owners sold off their flocks before our study got underway. Therefore, we replaced these six with an additional six randomly selected flocks to achieve our target of 62 flocks. We followed a two‐stage cluster sampling strategy: First, we randomly selected nomadic duck flocks from Mohanganj, and then we selected convenience samples of eggs from within the flock. The field team collected 30 egg samples from each flock's night shelter during early morning (usually around 6 am) of a day.

### 2.5 Duck flock owner interviews

Using a structured questionnaire, we interviewed all 62 flock owners and collected information about flock movement history, trading practices of ducks and eggs, contact with wild waterfowl, vaccination history, and flock biosecurity practices in the past year.

### 2.6 Sample collection

Field workers visited the night shelter of each nomadic duck flock in the early morning (usually around 6 am) for sampling and data collection. After obtaining informed consent from flock owners, field workers purchased 30 eggs laid that morning. Field workers also collected eight swab samples of pooled fresh fecal droppings from the four corners and four central positions of the night shelters and placed them in a single tube containing 5 mL of viral transport medium (VTM). Egg samples and pooled fecal samples were kept in a cold box maintained between 2°C and 8°C for 5‐7 days and transported to the BSL‐2 animal specimen laboratory at icddr,b.

### 2.7 Preparation of egg yolk and pooled fecal samples

Eggs were individually cracked and the egg white separated from the yolk using a sterile egg yolk separator. The yolk sac was ruptured with a needle and 4 mL of yolk was collected with a syringe under sterile conditions. Then, the yolk was mixed with an equal volume of 0.01 mol/L phosphate‐buffered saline (PBS; pH 7.2) and homogenized. The mixture was left for 1 hour at room temperature followed by centrifugation at 1500 *g* for 30 minutes. The supernatant (1.5 mL) was collected in Eppendorf tubes and stored at −20°C until testing. Pooled fecal samples were aliquoted in a tubes containing 1.8 mL VTM.

### 2.8 Laboratory methods

#### 2.8.1 H5 antibody detection by competitive enzyme‐linked immunosorbent assay (cELISA)

We tested egg yolk specimens to detect antibodies against avian influenza A (H5) using commercially available cELISA (AniGen H5 AIV Ab ELISA kit; BioNote, Gyeonggi‐do, South Korea). The kit uses a recombinant H5 hemagglutinin (HA) antigen that the manufacturer reports detects antibodies against avian influenza A (H5) in specimens with a higher sensitivity (100%) and specificity (99.9%) compared with hemagglutination inhibition (HI) assay (AniGen H5 AIV Ab ELISA kit; BioNote, Gyeonggi‐do, South Korea). The assay was performed according to the manufacturer's instructions (AniGen H5 AIV Ab ELISA kit; BioNote, Gyeonggi‐do, South Korea).The cELISA assay used in this study had 100% sensitivity and 96% specificity with egg yolk samples against H5N3 (A/wild bird feces/Korea/CSM2/2002 (H5N3) strain) compared with the hemagglutination inhibition assay.^24^ The cELISA and hemagglutination inhibition (HI) tests to detect avian influenza A virus antibodies in duck eggs had a good inter‐rater agreement (kappa) between tests (K>0.9).^24^ To classify the duck eggs as positive or negative, we used the manufacturer recommended cutoff value; percent inhibition (PI) values ≥75 were considered as positive and PI ≤75 as negative (AniGen H5 AIV Ab ELISA kit; BioNote, Gyeonggi‐do, South Korea).

#### 2.8.2 Detection of influenza A RNA by real‐time reverse‐transcriptase polymerase chain reaction (rRT‐PCR)

From the fecal swabs, we extracted viral nucleic acid using InviMag virus DNA/RNA mini kit KF96 (Stratec Molecular, Germany) and an automated processing system (KingFisher Flex Magnetic Particle Processor, Thermo Fisher Scientific, Waltham, MA, USA) following the manufacturer's instructions. We performed one‐step rRT‐PCR to screen for influenza A virus by targeting the matrix (M) gene, and all influenza A‐positive samples were further subjected to rRT‐PCR for H5 subtyping using H5a‐ and H5b‐specific primers and probes as previously described.^31^ A sample was considered positive for detection of influenza A virus RNA if the cycle of threshold (*C*_t_) was lower than 40.^32^ We did not attempt to test for H9, H7, or other subtypes of influenza as our focus was on the H5 subtype which has occurred commonly in Bangladesh.

### 2.9 Data analysis

We calculated proportions and medians for reporting the variables related to duck flock‐level demographic characteristics and management practices. We estimated the proportion of fecal samples and flocks with influenza A virus RNA with a 95% confidence interval using a log linear model with flock‐level clustering effect adjustment through clustered sandwich estimate of standard error.^33^ We also estimated the proportion of eggs containing antibodies against avian influenza A (H5) virus after taking into account the sensitivity (100%) and specificity (91%) of the cELISA test.^34^

### 2.10 Ethical considerations

We obtained informed consent from the owners of the nomadic duck flocks that were surveyed and sampled. We paid approximately eight Bangladeshi Taka (BDT) for the duck egg depending on the market value. The study protocol was reviewed and approved by the Ethical Review Committee (ERC) and Animal Experimentation Ethical Committee (AEEC) of icddr,b Bangladesh. We also received CDC Institutional Review Board (IRB) approval.

## 3 Results

### 3.1 Demographic characteristics of nomadic duck flocks

The median age of the ducks was 24 months (range: 8‐36 months). The median flock size was 300 ducks (range: 105‐1100). The median number of eggs produced daily by each flock was 160 (range: 150‐1100). The majority (63%) of flocks consisted of two breeds (Khaki Campbell and a local indigenous breed).

### 3.2 Nomadic duck raising practices

#### 3.2.1 Movement practices

Most flocks (98%) stayed within the scavenging area for a median time of 30 days (range: 15‐99). All flocks stayed in temporary confinement (made from bamboo skirt) during the night in the highlands near the scavenging area (Figure 3). All flock owners reported that scarcity of feed was the reason for moving flocks from one scavenging area to another. The median distance that flocks moved in a year was seven kilometers (range: 1‐150). Most owners moved flocks outside of their home village (87%) and most (79%) reported that they led the ducks on foot, while 16% transported ducks by boat and 5% by motor vehicle.

#### 3.2.2 Marketing practices

Most of the owners (90%) sold their entire flock when their egg production decreased. All duck eggs were sold in the village market in the flock owners’ subdistricts (100% N=62). Most of the ducks (92%) were sold to vendors and the remaining ducks (8%) were sold directly to retail customers (Table 1).

**Table 1:** Management practices of nomadic duck flocks reported by flock owners in northeastern Bangladesh, 2011‐2012

Nomadic duck flock management practices	N=62n (%)
Movement practices	
Longest distance (in kilometers) of movement of duck flocks in the past year, median (range)	7 (1‐150)
Places of movement of duck flocks in the past year	
Within own village	8 (13)
Into another village	36 (58)
Into another subdistrict	10 (16)
Into another district	8 (13)
Marketing practices	
Reasons for selling duck flocks	
Decreased egg production	56 (90)
Scarcity of feed	5 (8)
Disease outbreak(s)	1 (2)
Methods of selling duck flocks	
Vendors came to herding places to purchase	57 (92)
Brought to market	5 (8)
Method of selling duck eggs	
Brought the eggs to village markets	62 (100)
Biosecurity/biosafety practices	
Kept duck flocks away from chickens	7 (11)
Used disinfectant in night shelters	10 (16)
Took measures to prevent duck flocks from mixing with wild waterfowl in common feeding grounds	5 (8)
Methods of disposal of dead ducks	
Throwing into a water body	31 (50)
Burying	29 (47)
Burning	2 (3)
Reported hand washing techniques after collecting eggs	
With water alone	48 (77)
With soap and water	13 (21)
With ash	1 (2)
Vaccinated of flock for either duck plague or cholera	36 (58)

#### 3.2.3 Biosecurity practices

Most owners (71%) did not clean fecal droppings from the night shelter. A small number of owners (16%) used disinfectant in the night shelter. Half of the owners (50%) disposed of dead ducks by throwing them into adjacent water bodies. Almost all the owners (94%) reported that their duck flocks cofed with wild waterfowl. The majority of owners (58%) vaccinated their flocks against either duck plague or duck cholera. All owners reported that they did not vaccinate their duck against avian influenza A (H5N1). Most of the owners (77%) reported that they washed their hands with water from the nearby water bodies after collecting eggs. A few flock owners (11%) took measures to keep duck flocks away from chickens (Table 1). All duck flocks appeared healthy during sample collection.

### 3.3 Proportion of anti‐H5 antibodies in the eggs of nomadic duck flocks

Of the 1860 egg yolk samples collected, 556 (30%, 95%, CI: 28‐32) had H5 antibodies. Fifty‐eight flocks out of 62 (94%, 95% CI: 84‐98) had at least one egg with H5 antibodies. About half (47%) of the H5 antibody‐positive samples (261/556) had higher (≥90) percent inhibition values.

### 3.4 Proportion of influenza A virus RNA in the nomadic duck flocks

Of the 496 pooled fecal samples, 121 (24%, 95% CI: 22‐29) samples had detectable influenza A virus RNA by rRT‐PCR with a mean cycle threshold (*C*_t_) value of 36 (range: 24.9‐39.9), but none of the tested samples had detectable influenza type A (H5) RNA. Thirty‐three flocks (53%, 95% CI: 40‐66) had at least one pooled fecal sample that tested positive for influenza A virus RNA.

## 4 Discussion

The study provides molecular evidence of influenza A and antibody evidence of avian influenza A (H5) virus infections among nomadic ducks in northeastern Bangladesh. Nomadic duck raising activities provide important financial support to the duck owner's families, and low‐lying areas with large bodies of water are a favorable environment for nomadic duck raising. However, nomadic ducks are exposed to H5 influenza viruses and are substantial reservoirs of avian influenza A viruses in northeastern Bangladesh.

In this study, egg yolk samples had a much higher proportion (30%) of antibodies against avian influenza A (H5) virus in nomadic ducks than the reported proportion of antibodies detected from blood samples in studies from Indonesia (3%)^6^ and Vietnam (18%).^8^ The higher prevalence of antibodies may reflect higher exposure to H5N1 in this region of Bangladesh compared to the studied regions in Indonesia and Vietnam, although the age of the ducks may also have contributed to the high levels of seroprevalence. Most (90%) of the ducks studied in Bangladesh were adult (>12 months). One study found that adult ducks had higher seroprevalence of influenza A virus than subadults (<12 months) in Vietnam,^8^ presumably because of more opportunities for repeat exposure to influenza A viruses as a duck ages.^35^ Another possible explanation for these findings is that the ducks could have been exposed to avian influenza A (H5) virus which was circulating in this region a few months prior to our study.^36^

Nomadic ducks contact with wild waterfowl in the bodies of water during the winter months and contact with other poultry species and humans in the duck owner villages during the summer months. This may pose an increased risk of interspecies transmission of avian influenza A viruses in Bangladesh compared with Thailand, Indonesia, and Vietnam (Table S1).^6^, ^7^, ^9^ Infected wild waterfowl carry avian influenza A viruses and may spread them along their migratory route introducing these viruses into the poultry flocks.^37^ Our study shows that nomadic ducks were infected with influenza A viruses. Flock owners reported interaction between nomadic ducks and wild waterfowl during the winter period (November‐February) and nomadic ducks had close contact with backyard chickens and humans while staying in the owners’ home villages during the summer months (March‐June). Nomadic ducks in Bangladesh may serve as a bridging host for interspecies transmission of avian influenza A viruses from wild water fowl to backyard poultry or vice versa. Interspecies transmission is a public health concern because of the potential for viral adaption or reassortment between viruses affecting these varied hosts.^38^

Nomadic duck raising practices were characterized by movement outside of the owners’ home villages, transporting duck flocks on foot and marketing ducks and their eggs. This could contribute to regional spreading of avian influenza A viruses when nomadic ducks are actively shedding virus.^5^, ^6^, ^7^, ^9^ The practices and levels of infection reported in this study may help inform modeling efforts describing the potential bidirectional spread of avian influenza A viruses between wild waterfowl, nomadic ducks, and domestic poultry in Bangladesh.^39^

More than one quarter of nomadic duck flocks in our study shed influenza A viral RNA into the environment from their fresh fecal droppings, which is comparable to other studies.^3^, ^4^ Duck flocks that shed influenza A viruses while appearing healthy are also consistent with other studies.^3^, ^6^

We did not detect any H5 virus RNA in environmental fecal samples of nomadic duck flocks during our study period. Several factors may have contributed to this observation. The low nucleic acid content (mean *C*_t_ value was 36) among influenza A‐positive samples provided low sensitivity to detect H5. There may have been no active H5 circulation at the time of our study. Many of the nomadic ducks in our study may have been immune to avian influenza A (H5) infection due to previous exposure as indicated by high (30%) antibody positivity against avian influenza A (H5) virus.

**Figure 2:**
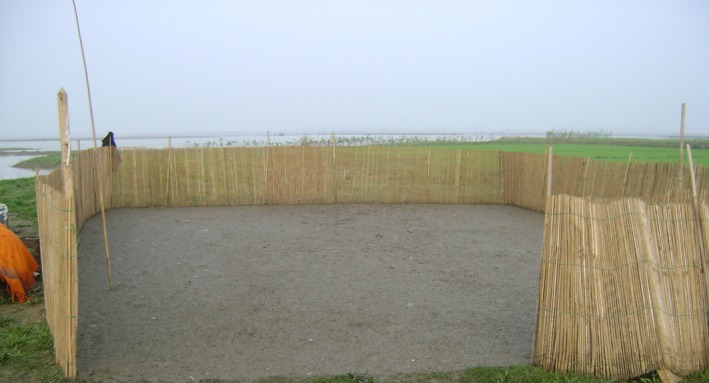
A nomadic duck night shelter in study area

**Figure 3:**
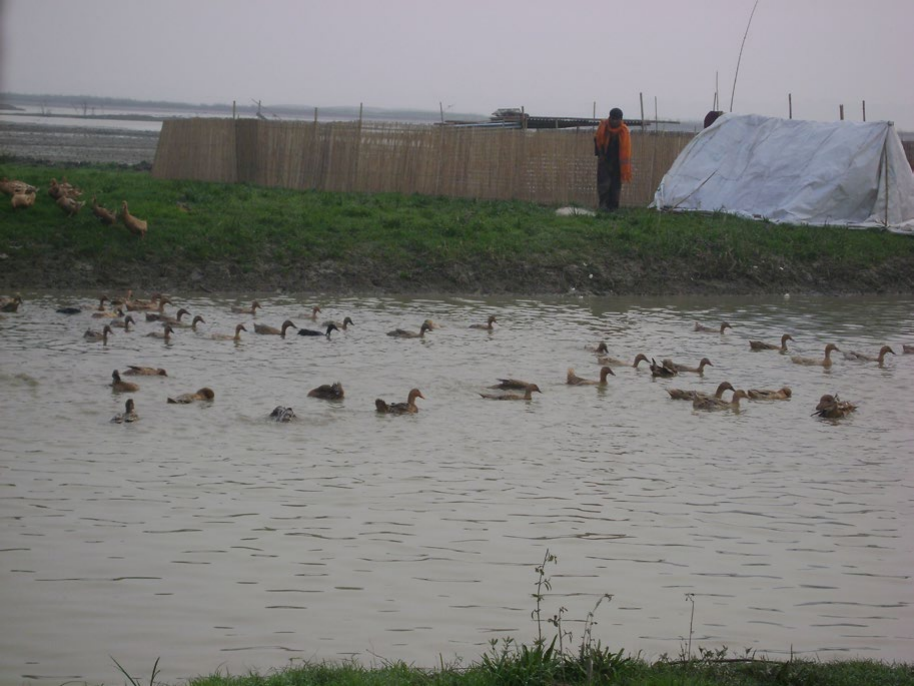
A nomadic duck flock in the bodies of water and a night shelter

Few flock owners reported cleaning fecal material or disinfecting their duck night shelters (Figures 2 and 3). Duck night shelters do not have a floor so duck feces remain on the ground after the shelter is relocated. However, fecal contamination from night shelters may contribute to influenza A virus maintenance in the environment as well as infection of other ducks within the flock. A study in Cambodia suggests that influenza A virus‐contaminated environmental materials may act as potential sources for human and/or animal infection.^40^ Moreover, the shelters have sides, which made from bamboo skirts could act as vehicles for AIV transmission to another location, because they frequently soiled with duck feces and not properly cleaned.

Half of the duck flock owners reported that they disposed of dead ducks by throwing them into adjacent water bodies, which is similar to other studies conducted in Bangladesh.^41^ If ducks are infected, this practice may also spread influenza A viruses in the environment.

Limited biosecurity and hygiene practices may also contribute to the risk of interspecies transmission of avian influenza A viruses including gene exchange of different subtypes of influenza A viruses between nomadic ducks, wild waterfowl, chickens, and possibly humans.

The study has some limitations. First, we examined duck flocks in only one subdistrict so the findings are not statistically representative of all nomadic duck raising areas in Bangladesh. However, this is a major duck raising area and represents a substantial risk for avian influenza virus transmission. Second, we conducted the study during a short period in the winter and exposure to, and shedding of, influenza A viruses may be subject to seasonal variations.^21^, ^28^ Nevertheless, the study was conducted during peak season for avian influenza A (H5) virus circulation in Bangladesh.^42^ Third, instead of structured observation, we depended on reports from duck raisers to describe duck raising practices over the past year which may have been affected by social desirability bias 43 and so the reports of high risk behavior should be seen as minimal estimates. Fourth, we utilized cELISA kit to detect antibodies against avian influenza A (H5), which does not distinguish highly pathogenic from low pathogenic H5 strains. However, widespread outbreaks of highly pathogenic H5N1 in Bangladesh^22^, ^44^, ^45^ since 2007, including HPAI H5N1 outbreak among waterfowl that reported in the study area before 5 months of this study,^22^ suggest that widespread infection with low pathogenic H5 infections is an unlikely explanation for these results.

Nomadic duck raising is the primary livelihood for the low‐income nomadic duck owners in our study. Investments in improved hygiene and biosecurity measure risk being unaffordable. To develop an affordable and effective intervention, it is important to understand the duck flock owners’ perspectives to identify which practices to target and how to change these practices.^46^ Interventions to change behavior are more likely to be successful when aligned with the financial incentives of the target population.^47^, ^48^ Specifically, biosecurity interventions that cost effectively improve duck survival and egg production are probably more likely to be adopted. We recommend further research to develop and evaluate interventions that simultaneously improve duck raisers profitability and biosecurity.
