# Supplementary material for: An epidemiological study of avian influenza A (H5) virus in nomadic ducks and their raising practices in northeastern Bangladesh, 2011‐2012
Source: Influenza Other Respir Viruses. 2017 Jan 2;11(3):275–82. doi: 10.1111/irv.12438 (PMC5410719; doi:10.1111/irv.12438)
Supplement: Supplementary file 1 [file IRV-11-275-s001.doc]

**Table S1:** Comparison of duck raising practices among Thailand, Indonesia, Vietnam and Bangladesh

| **Variable** | **Thailand** | **Indonesia** | **Vietnam** | **Bangladesh ( from this study)** |
| --- | --- | --- | --- | --- |
| Feeding site | Duck flocks feed year round in wetland used for double crop rice production | Duck flock graze in the rice field | Duck flocks feed in rice field after harvest to scavenge for leftover rice grains | Ducks feed in large water bodies during winter period (Oct-Feb);  During summer season (March- September), ducks staying owners' villages |
| Movement | Frequent rotation of duck flocks in rice paddy fields after the harvest | Allowed to scavenge freely during the day around houses, in the villages or in rice paddies | Duck flock moves within or between districts and/or provinces throughout the year | Duck flocks move from one location to another for feed (Oct-Feb) |
| Duck shelter | Duck shelters located within villages | Duck shelters located within villages | Duck shelters located within villages | Ducks shelters are located near large water bodies/scavenging areas |
| Flock size (mean) | NA | 53.7 | 400 | 300 |
| Duck production cycle | The duck production cycle is closely connected with rice crops because rice provides duck feed. | The duck production cycle is closely connected with rice crops because rice provides duck feed. | The duck production cycle is closely connected with rice crops because rice provides duck feed. | The duck production cycle is closely connected with large water bodies in the low laying areas- the water bodies provide feed |
| Total duck | 69 million | NA | 33 million | 41 million |

^*^NA Not available
