# Supplementary material for: An epidemiological study of avian influenza A (H5) virus in nomadic ducks and their raising practices in northeastern Bangladesh, 2011‐2012
Source: Influenza Other Respir Viruses. 2017 Jan 2;11(3):275–82. doi: 10.1111/irv.12438 (PMC5410719; doi:10.1111/irv.12438)
Supplement: Supplementary file 2 [file IRV-11-275-s002.doc]

| Specificity | Primer/probe | Sequence (5'-3') | Remarks |
| --- | --- | --- | --- |
| H5 | Inf_H5a_Forward | 5’-TGG AAA GTR TAA RAA ACG GAA CGT-3’ |  |
|  | Inf_H5a_Reverse | 5’-YGC TAG GGA RCT CGC CAC TG-3’ |  |
|  | Inf_H5a_Probe 2 2 | 5’-FAM-CAA CTA TCC GCA G**"T"**A TTC AGA AGA AGC AAG ATT AA-3’ | Quenched internally at a modified **“T” residue with BHQ1** and a **terminal phosphate at the 3’-end** to prevent probe extension by Taq polymerase. |
|  | Inf_H5a_Probe 1 2 | 5’-FAM-TGA CTA CCC GCA G**"T"**A TTC AGA AGA AGC AAG ACT AA-3’ |  |
|  | Inf_H5b_Forward | 5’-GGA ATG YCC CAA ATA TGT GAA ATC AA-3’ |  |
|  | Inf_H5b_Reverse | 5’-CCA CTC CCC TGC TCR TTG CT-3’ |  |
|  | Inf_H5b_Probe2 | 5’-6-FAM-TAC CCA TAC CAA CCA **"T"**CT ACC ATT CCC TGC CAT-3’ | Quenched internally at a modified **“T” residue with BHQ1** and a **terminal phosphate at the 3’-end** to prevent probe extension by Taq polymerase. |

Table S2: Primers and probes designed for influenza A (H5) specific real-time RT-PCR
